# Stereotactic Cortical Atlas of the Domestic Canine Brain

**DOI:** 10.1038/s41598-020-61665-0

**Published:** 2020-03-16

**Authors:** Philippa J. Johnson, Wen-Ming Luh, Benjamin C. Rivard, Kathleen L. Graham, Andrew White, Marnie Fitz-Maurice, John P. Loftus, Erica F. Barry

**Affiliations:** 1 Cornell College of Veterinary Medicine, Department of Clinical Sciences, Cornell University, Ithaca, NY USA; 2 National Institute of Aging, National Institutes of Health, Baltimore, MD USA; 3 Clinical Ophthalmology and Eye Health, Sydney Medical School, University of Sydney, Sydney, NSW Australia; 4 Cornell College of Veterinary Medicine, Department of Biomedical Sciences, Cornell University, Ithaca, NY USA

**Keywords:** Computational neuroscience, Brain

## Abstract

The domestic canine (*canis familiaris*) is a growing novel model for human neuroscientific research. Unlike rodents and primates, they demonstrate unique convergent sociocognitive skills with humans, are highly trainable and able to undergo non-invasive experimental procedures without restraint, including fMRI. In addition, the gyrencephalic structure of the canine brain is more similar to that of human than rodent models. The increasing use of dogs for non-invasive neuroscience studies has generating a need for a standard canine cortical atlas that provides common spatial referencing and cortical segmentation for advanced neuroimaging data processing and analysis. In this manuscript we create and make available a detailed MRI-based cortical atlas for the canine brain. This atlas includes a population template generated from 30 neurologically and clinically normal non-brachycephalic dogs, tissue segmentation maps and a cortical atlas generated from Jerzy Kreiner’s myeloarchitectonic-based histology atlas. The provided cortical parcellation includes 234 priors from frontal, sensorimotor, parietal, temporal, occipital, cingular and subcortical regions. The atlas was validated using an additional canine cohort with variable cranial conformations. This comprehensive cortical atlas provides a reference standard for canine brain research and will improve and standardize processing and data analysis and interpretation in functional and structural MRI research.

## Introduction

There is continual need to develop novel animal models for neurobiological and neuropsychological research. The domestic canine (*canis familiaris*) shows multiple advantages over more standard rodent and primate models and there is growing use of the dog as a model in neurocognitive, aging and clinical research. Unlike rodents and primates, dogs are highly-trainable and able to undergo non-invasive experimental procedures without restraint, including functional magnetic resonance imaging (fMRI)^1,2^. In addition, the canine brain has the advantage of being gyrencephalic, making it more similar to the human brain than rodent and avian models. Neurocognitively the canine shares similar behavioral and emotional responses to humans and are highly integrated into human society. These convergent sociocognitive skills places the dog in a unique position to increase our understanding of sociocognition in humans^3^. The aging canine is being routinely used as model for aging research due to its unique similarities to human brain aging and ability to link aging with learning memory and other cognitive functions^4–7^. The canine also suffers from some spontaneous neurological diseases analogous to that of humans, and as such can serve as a unique model for these disease processes including glioma^8^ and amyotrophic lateral sclerosis^9^. This growing use of the dog in non-invasive neuroscience, aging and neuropathogical research has generated a need for a standard canine brain atlas that provides common spatial referencing and architectonic based cortical segmentation for standardized data processing, analysis and interpretation^3^.

Several brain atlases have been made available for the canine^10–12^, however these atlases have limitations, being created from a low number of subjects^10^, using non-isovolumetric clinical magnetic resonance imaging (MRI) data^12^, or utilizing dogs that were not neurologically or clinically healthy^11^. In addition, there is no cortical atlas that provides a microarchitectonic based cortical parcellation for the canine brain^12^. Cortical brain atlases allow for standardized referencing of brain regions within a particular species and assist in the correlation of function and structural brain regions between species. Digital cortical atlases can be viewed 3-dimensionally and can be used for computational processing and transformation, a critical component for quantitative analysis of MRI data^13^.

Atlases of the cerebral cortex have been historically created by partitioning into regions with distinct laminar structures using histologically defined criteria. The most commonly used human MRI cortical atlases were created based on cytoarchitectonic maps created by the German anatomist Korbinian Brodmann^14^ which separated areas of the cortex according to cytoarchitectural organization. Although used commonly, there is a concern that these atlases do not provide sufficient neuroanatomical detail for the degree of cortical segregation more recently identified in neuroimaging research^15,16^. Though more fine-grained cytoarchitectonic atlases exist, such as Economo and Koskinas, 1925 atlas^17^ and Sarkisov, 1949 atlas^18^ they have not been widely utilized. For this reason there has been growing, interest in using a different component of neuronal organization, myeloarchitecture, to create a human cortical atlases such as the one generated by anatomists Oskar and Cecile Vogt^16,19,20^ and Flechsig^21^. The atlases by Vogt^17,20,21^ divide the cortex according myeloarchitecture using the density, orientation and configuration of myelinated axons resulting in the division of the human cortex into 185 regions. These regions are thought to be complementary to cytoarchitectonic based cortical divisions. Currently, a “supermap” of the human neocortex is being created using myeloarchitectonics from the Vogt-Vogt School and has the potential to be a tool that is more detailed and morphologically more accurate than currently available cytoarchitectonic atlases^16^. Similarly research by Walters^22^ have shown have shown a direct correlation between the myeloarchitecture of the human cortex and MRI signal intensities could be applied to other species.

The canine cortex has been intricately studied by Jerzy Kreiner who generated a comprehensive myeloarchitectonic-based cortical atlas^23–28^. These document the parcellation of the cortex according to the size, staining, appearance, and arrangement of radial and tangential myelinated fibers and the appearance of myelinated fibers in the superficial plexus^24^. These manuscripts provide detailed surface and cross-sectional illustrations to show the exact margins of each region, facilitating segmentation^23–28^. They intricately segment the cortex into regions, similar to that described by the Vogt-Vogt school^16^.

In this manuscript we create a stereotactic cortical atlas for the mesaticephalic canine brain based on data from Kreiner’s myeloarchitectonic parcellations. This cortical atlas is created with a population average template generate from high-resolution 3-dimensional T1-weighted data obtained from 30 neurologically normal dogs. This quality assured and validated atlas includes tissue segmentation maps and a total of 234 cortical and subcortical priors. The atlas is provided in common neuroimaging informatics technology initiative (NIfTI) format and can be integrated into standard neuroscience tools and pipelines for data analysis and processing. This comprehensive cortical atlas provides a reference standard for canine brain research and will improve and standardize processing and data analysis and interpretation in functional and structural MRI research.

## Materials and Methods

### Study population

For template creation, we recruited 30 dogs from research populations (Cornell University College of Veterinary Medicine). In order to limit the diversity of brain structure between subjects secondary to cranial conformation, we included only non-brachycephalic dogs considered clinically and neurological normal. The population was composed of 22 females and 8 males aged between 2 and 11 years of age (median 5.5, interquartile range 7.5). Ten of these subjects were beagles and twenty were of mixed breed, weighing between 7 and 30 kgs (median 13, interquartile range 12.75). All dogs were imaged for research purposes and the Cornell University Institutional Animal Care and Use Committee (IACUC protocol number: 2015–0115) approved their use (Table 1). All procedures were performed in accordance with the relevant guidelines and regulations.

**Table 1:** Signalment and brain characteristics of subjects included in the final template. F = female, Fs = female spayed, M = male, Mn= male neutered.

Subject	Breed	Sex	Age (years)	Weight (kg)	Brain length	Brain width	Cephalic index	Cranial conformation
1	Beagle	F	2	9	6.93	5.01	72.29	Masticephalic
2	Beagle	F	2	9	6.99	5.22	74.68	Masticephalic
3	Beagle	Fs	2	7	7.18	5.14	71.59	Masticephalic
4	Beagle	Fs	2	9	7.2	5.16	71.67	Masticephalic
5	Beagle	M	7	9	7.22	5.12	70.91	Masticephalic
6	Beagle	F	2	7	7.26	5.04	69.42	Masticephalic
7	Mixed breed	F	6	11	7.33	5.11	69.71	Masticephalic
8	Beagle	F	2	9	7.34	4.85	66.08	Masticephalic
9	Beagle	Fs	5	9	7.45	5.33	71.54	Masticephalic
10	Beagle	F	2	8	7.47	5.15	68.94	Masticephalic
11	Mixed breed	F	6	12	7.65	5.06	66.14	Masticephalic
12	Mixed breed	F	6	14	7.69	5.2	67.62	Masticephalic
13	Mixed breed	F	4	15	7.79	5.33	68.42	Masticephalic
14	Mixed breed	Fs	11	21	7.83	5.41	69.09	Masticephalic
15	Beagle	F	2	9	7.93	5.28	66.58	Masticephalic
16	Mixed breed	F	5	10	7.94	5.21	65.62	Masticephalic
17	Mixed breed	F	11	20	8.05	5.19	64.47	Masticephalic
18	Mixed breed	F	6	12	8.15	5.19	63.68	Masticephalic
19	Mixed breed	F	5	12	8.15	5.28	64.79	Masticephalic
20	Mixed breed	Fs	11	22	8.4	5.67	67.50	Masticephalic
21	Mixed breed	Mn	4	18	8.41	5.51	65.52	Masticephalic
22	Mixed breed	M	5	28	8.52	5.4	63.38	Masticephalic
23	Mixed breed	M	10	29	8.57	5.41	63.13	Masticephalic
24	Mixed breed	Fs	10	22	8.6	5.47	63.60	Masticephalic
25	Mixed breed	M	10	24	8.72	5.59	64.11	Masticephalic
26	Mixed breed	Fs	10	20	8.85	5.58	63.05	Dolichocephalic
27	Mixed breed	Fs	10	29	8.97	5.63	62.76	Dolichocephalic
28	Mixed breed	Mn	5	30	8.99	5.85	65.07	Dolichocephalic
29	Mixed breed	M	10	20	9.11	5.41	59.39	Dolichocephalic
30	Mixed breed	M	11	31	9.56	5.7	59.62	Dolichocephalic

For skull conformation compatibility testing, data sets from twelve dogs were recruited from a neurologically normal clinical research population (University of Sydney College of Veterinary Science). Five subjects were clinically healthy and seven were previously diagnosed with glaucoma affecting a single or both eyes. All dogs were female aged between 5 and 11 years of age (median 9, interquartile range 3.5). The cohort weighed between 4.7–35.3 kg (median 8.4, interquartile range 7.48) and included the following breeds, flat-coat retriever (n = 1), cocker spaniel (n = 2) and cattle dog (n = 1), Maltese crossbreed (n = 3), labradoodle (n = 3) and terrier crossbreed (n = 2) (Table 2). All dogs were imaged for research purposes and the University of Sydney Ethics Committee approved their use (Protocol no. 2017/1156).

**Table 2:** Signalment and brain characteristics of subjects included in the testing cohort. Fs = female spayed.

Subject	Breed	Sex	Age (years)	Weight (kg)	Brain length	Brain width	Cephalic index	Cranial conformation
1	Terrier mixed breed	Fs	6	7	5.49	4.91	89.44	Brachycephalic
2	Maltese mixed breed	Fs	11	5	5.93	4.96	83.64	Brachycephalic
3	Terrier mixed breed	Fs	6	7	5.95	5.07	85.21	Brachycephalic
4	Maltese mixed breed	Fs	11	5	6.02	4.87	80.90	Brachycephalic
5	Labradoodle	Fs	9	8	6.39	4.65	72.77	Brachycephalic
6	Maltese mixed breed	Fs	11	7	6.58	4.85	73.71	Brachycephalic
7	Labradoodle	Fs	9	9	6.62	4.75	71.75	Brachycephalic
8	Labradoodle	Fs	10	14	7.65	5.39	70.46	Mesaticephalic
9	Cattle Dog	Fs	7	19	7.66	5.34	69.71	Mesaticephalic
10	Cocker Spaniel	Fs	10	14	7.7	5.34	69.35	Mesaticephalic
11	Cocker Spaniel	Fs	9	15	8.27	5.74	69.41	Mesaticephalic
12	Flat-coat Retriever	Fs	5	35	9.42	5.85	62.10	Dolichocephalic

#### MRI examination

Dogs imaged for template creation were imaged under general anesthesia performed by a board-certified veterinary anesthesiologist. Dogs were premedicated with dexmedetomidine (3 mcg/kg Dexdomitor 0.5 mg/ml, Zoetis Inc, Kalamazoo, MI), induced to general anesthesia with propofol to effect (3.2–5.4 mg/kg Sagent Pharmaceuticals, Schaumburg, III) and intubated. They were maintained under anesthesia with inhalant isoflurane and oxygen with a dexmedetomidine continuous rate infusion (1 mcg/kg/hr Dexdomitor 0.5 mg/ml, Zoetis Inc, Kalamazoo, MI). MRI was performed in a 3.0T General Electric (GE) Discovery MR750 (GE Healthcare, Milwaukee, WI) whole body scanner (60 cm bore diameter), operating at 50mT/m amplitude and 200T/m/s slew-rate. Subjects were placed in dorsal recumbency with their head centered in a 16-channel medium flex radio-frequency coil (NeoCoil, Pewaukee, WI 53072 USA). A high-resolution T1-weighted 3D inversion-recovery fast spoiled gradient echo sequence (Bravo) was performed in each subject with the following parameters; isotropic voxels 0.5 mm^3^, TE = 3.6 ms, TR = 8.4 ms, TI = 450 ms, excitations = 3, a flip angle of 12°, acquisition matrix size = 256 ×256.

Dogs imaged for skull shape compatibility validation were imaged under general anesthesia performed by a trained veterinary anesthesiologist. All animals were premedicated with methadone (0.1–0.4 mg/kg IM; Physeptone, Aspen Pharma Pty Ltd, St Leonards NSW) with or without acepromazine (0–0.03 mg/kg IM; ACP-2, Ceva Animal Health Pty Ltd, Glenorie NSW). General anesthesia was induced with propofol (4–6 mg/kg IV; Propofol, Sandoz Pty Ltd, Pyrmont NSW) or thiopentone (4 mg/kg IV; Pentothal, Link Medical Products Pty Ltd, Warriewood NSW) to effect and intubated. Inhalational isoflurane and oxygen maintained general anesthesia. Imaging was performed in a 3.0T GE Discovery MR750 (GE Healthcare, Milwaukee, WI) whole body scanner using an 8-channel extremity coil (HD Foot Ankle array, Invivo) with the dog positioned in dorsal recumbency. A T1-weighted 3D fast spoiled gradient recalled echo (FSPGR) pulse sequence was performed with the following parameters; isotropic voxels 0.6 mm^3^, TE = 2.8 ms, TR = 6 ms, TI = 450 ms, excitations = 1, flip angle = 12°, acquisition matrix size = 192 × 192, slice thickness = 0.6 mm.

### Data processing

#### Preprocessing

**Figure 1:**
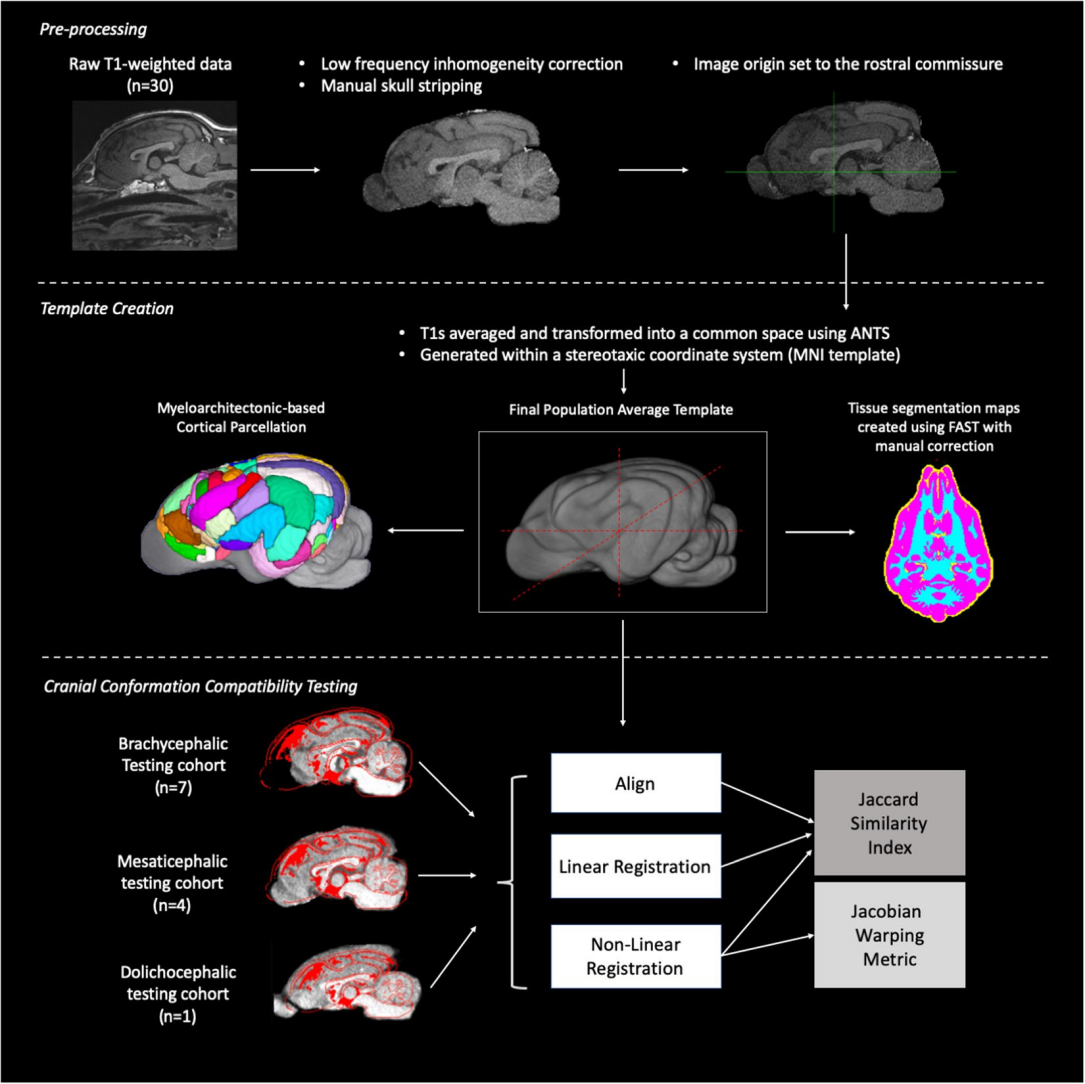
Method flow chart: Flow chart demonstrating the pre-processing, template creation and cranial conformation compatibility testing steps that were performed. (n = number of subjects, ANTs = advanced normalization tools, FAST = FMRIB’s automated segmentation tool, MNI = Montreal Neurological Institute). This figure was created using FSLeyes (version 2.1 https://fsl.fmrib.ox.ac.uk/fsl/fslwiki/FSLeyes), OsiriX MD (version 11.0 https://www.osirix-viewer.com/osirix/osirix-md/) and Microsoft Powerpoint (version 16.16.19. www.microsoft.com).

Isovolumetric T1-weighted data from the template group were used to create a population average atlas template. MRI data were corrected for low-frequency inhomogeneity^29^. A manual removal of non-brain tissues was applied prior to registration and spatial normalization^30^. The origin of images were manually set to the rostral commissure using SPM12^31^ and reoriented to a standard FMRI Software Library (FSL) orientation for inter-subject consistency where the x-axis contains right-left orientation, the y-axis contains the caudal-rostral orientation and the z-axis contains the ventral-dorsal orienation^32^. A flow chart depicts the steps we undertook during data processing and template validation (Figure 1).

#### Template creation

Previous atlas literature have tested linear and non-linear methods for template creation and consistently found non-linear registration using Advanced Normalization Tools (ANTs) to provide templates with the best contrast and signal to noise ratios^12,33,34^. For this reason, we opted to use non-linear registration methods to create our population average template. The individual subjects T1s were averaged and transformed into a common space population template using Advanced Normalization Tools (ANTs) which applied affine registration and diffeomorphic registration via the symmetric normalization (SyN) algorithm using the ANTs multivariate template creation script (Avants *et al*.^35,36^, 2010). This template was generated with a stereotaxic coordinate system according to the Montreal Neurological Institute (MNI) template specifications and in line with other animal templates^12,37^. The origin of the Cartesian system (x,y,z; 0,0,0) was centered on the mid-line over the dorsal aspect of the rostral commissure. The zero x-axis value sagittal plane extended through the center of the brain in line with the falx cerebri, the zero y-axis value transverse plane was parallel to the anterior commissure and transected the brain symmetrically and the zero z axis value dorsal plane ran from the dorsal rostral commissure to the mesencephalic aqueduct, ventral to the caudal commissure. Sagittal plane x-axis values increased left to right, transverse plane y-axis values increased caudal to rostral and dorsal plane z-axis values increased ventral to dorsal. All co-ordinates are provided in millimeters. A neuroanatomical expert evaluated the final template and compared to anatomic specimens for appropriate anatomical detail. Tissue segmentation maps (TSMs) were created from the template using FMRIB’s Automated Segmentation Tool (FAST) which segments brain matter into cerebral spinal fluid (CSF), grey matter (GM), and white matter (WM) while correcting for spatial intensity variations^38^. FAST was used to create partial volume maps, TSMs of each tissue type, binary segmentation masks and bias field maps. These maps were evaluated and manually corrected to ensure anatomical coherence with the T1 weighted scan. The corrected partial volume masks were used to calculate the tissue volume to account for partial volume effects and increase sensitivity. Figure 1 documents the template creation steps undertaken.

#### Determination of cranial conformation

Canine cranial conformation is highly variable between animals of different breed and genetic make-ups. There is currently no clear consensus on how to categorize dogs into brachycephalic (short-faced), mesaticephalic (medium-faced) and dolichocephalic (long-faced) groups. Milne *et al*. (2016) explored multiple different techniques and found that brain length correlated most strongly with a subjective categorization of brain conformation. For this reason, we utilized brain length parameters to identify the cranial conformation of all subjects included in the brain template and testing cohorts. Data sets with a brain length <68 mm were classified as brachycephalic, 72–87 mm were classified as mesaticephalic and >88 mm were classified as dolichocephalic^11^. These measures confirmed that the template cohort included 25 mesaticephalic and five dolichocephalic subjects (Table 1) and the testing cohort included seven brachycephalic subjects, four mesaticephalic and one dolichocephalic (Table 2).

#### Skull conformation compatibility

In order to test the impact of registration on brains with differing cranial conformation the testing cohort, made up of five mesaticephalic, one dolichocephalic and seven brachycephalic subjects, were registered and assessed for similarity to the template using the Jaccard similarity index and warping using the Jacobian warping metric. Individual subject data were corrected for low-frequency inhomogeneity (Tustison *et al*.^29^) and manual removal of non-brain tissues was applied. Each subject’s brain data were registered to the population template using alignment (center of image 0,0,0 at the anterior commissure with anatomical alignment through the rostral commissure and ventral brain regions), rigid linear registration (registering each subject to the template with six degrees of freedom) using FMRIB’s Linear Registration Tool (FLIRT)^39^ and nonlinear registration using FMRIB’s Nonlinear registration (FNIRT)^40^. Binary brain masks were generated for each subject at each level of registration i.e. aligned mask, linear mask, and nonlinear mask.

#### Jaccard similarity index

The degree of similarity between the individual subject and template masks was tested using the Jaccard similarity index. The index was able to calculate the amount of overlapping between individual subjects at each level of registration compared to the template mask. The Jaccard similarity index between the masks (i.e., subject 1 aligned to template mask etc.) was calculated using the following commonly used formula:

$${Jaccard}\,Index=\frac{number\,of\,voxels\,in\,both\,sets}{number\,of\,voxels\,in\,either\,set}\ast 100$$

This measure of similarity was compared across skull shape groups and registration method to identify any significant differences between skull shape and similarity to the population template^41^. A one-way ANCOVA explored the differences between similarity metrics across registration techniques while controlling for interaction effects of body weight (kg), brain volume (mm^3^) and brain length. Similarly, an ANCOVA tested the differences in alignment similarity between brachycephalic and mesaticephalic groups while covarying for body weight (kg), brain volume (mm^3^) and brain length. Statistically significant differences or associations were considered present when p < 0.05.

#### Jacobian warping metric

In order to assess the degree of warping that each subject underwent during non-linear registration Jacobian determinants for each voxel were calculated as a measure of nonlinear warping. In order to visualize and explore the localization and pattern variation of warping across the dog cranial conformation groups, the log-demeaned absolute Jacobian warpfield images were tested for variation by one sample T-test using FSL’s *randomize* tool for permutation testing general linear models^42^ for each cranial conformation testing group, brachycephalic (n = 7) and mesaticephalic (n = 4). Since there was a single dolichocephalic subject, this group was not considered for testing. These permutations aim to test the null hypotheses that the mean variation is symmetrical and therefore centered around zero. The output t-statistic was corrected for multiple comparisons using threshold-free cluster enhancement and thresholded at p < 0.05 significance. A post hoc Tukey multiple comparisons of means at 95% family-wise confidence levels explored the differences between each registration method. Mean Jacobian warping metric for each subject across all voxels was plotted with each cranial conformation group. For visualization purposes three subjects’ (one brachycephalic, one mesaticephalic and one dolichocephalic) log demeaned Jacobian warpfields were presented in a 3D format to highlight regional variation across dogs of different skull shapes.

**Figure 2:**
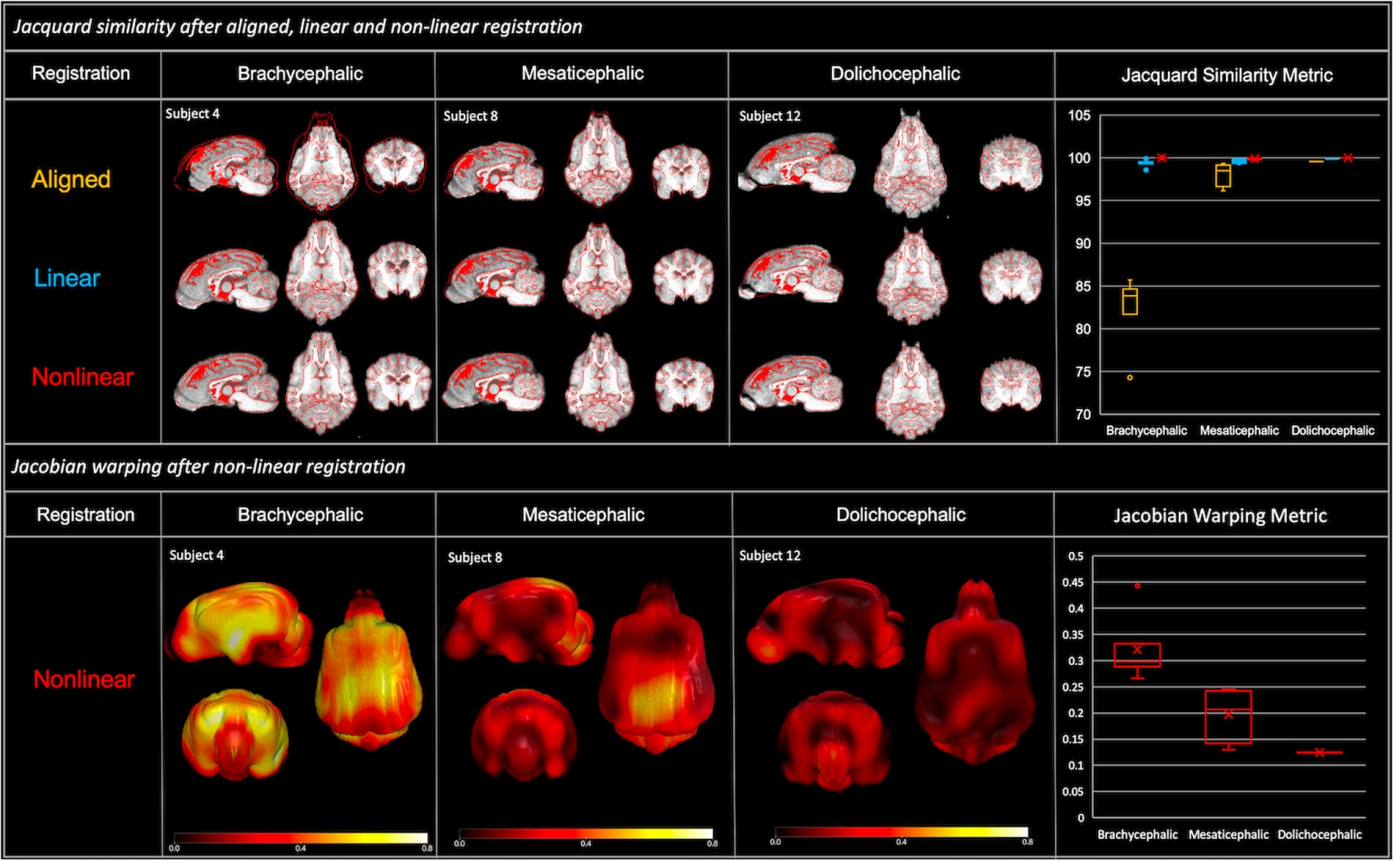
Gyral anatomy: Demonstrates the gyral surface anatomy of the final population average template and correlates that to a mesaticephalic anatomic specimen. The anatomic specimen underwent emersion fixation in 10% buffered formalin after removal from the cranium (g. = gyrus, cd. = caudal, rost. = rostral). This figure was created using FSLeyes (version 2.1 https://fsl.fmrib.ox.ac.uk/fsl/fslwiki/FSLeyes) and Microsoft Powerpoint (version 16.16.19. www.microsoft.com).

#### Cortical parcellation

Cortical parcellation into myeloarchitectonic regions was performed manually on the canine population template. Researchers divided the cortex into the following lobes; frontal, cingulate, parietal, sensori-motor, temporal (perisylvian) and occipital following the myeloarhitectonic articles from Jerzy Kreiner^23–28^. Lobe boundaries were established based on the demarcations in Kreiner’s articles. Within these lobes individual regions were parcellated based on Kreiner’s detailed descriptions and depictions of cortex surfaces, sagittal and transverse slices, and referencing histological atlases^43,44^. In total, 234 regions were parcellated by trained researchers (EFB and BR) and reviewed by a canine MRI anatomy expert (PJJ).

**Figure 3:**
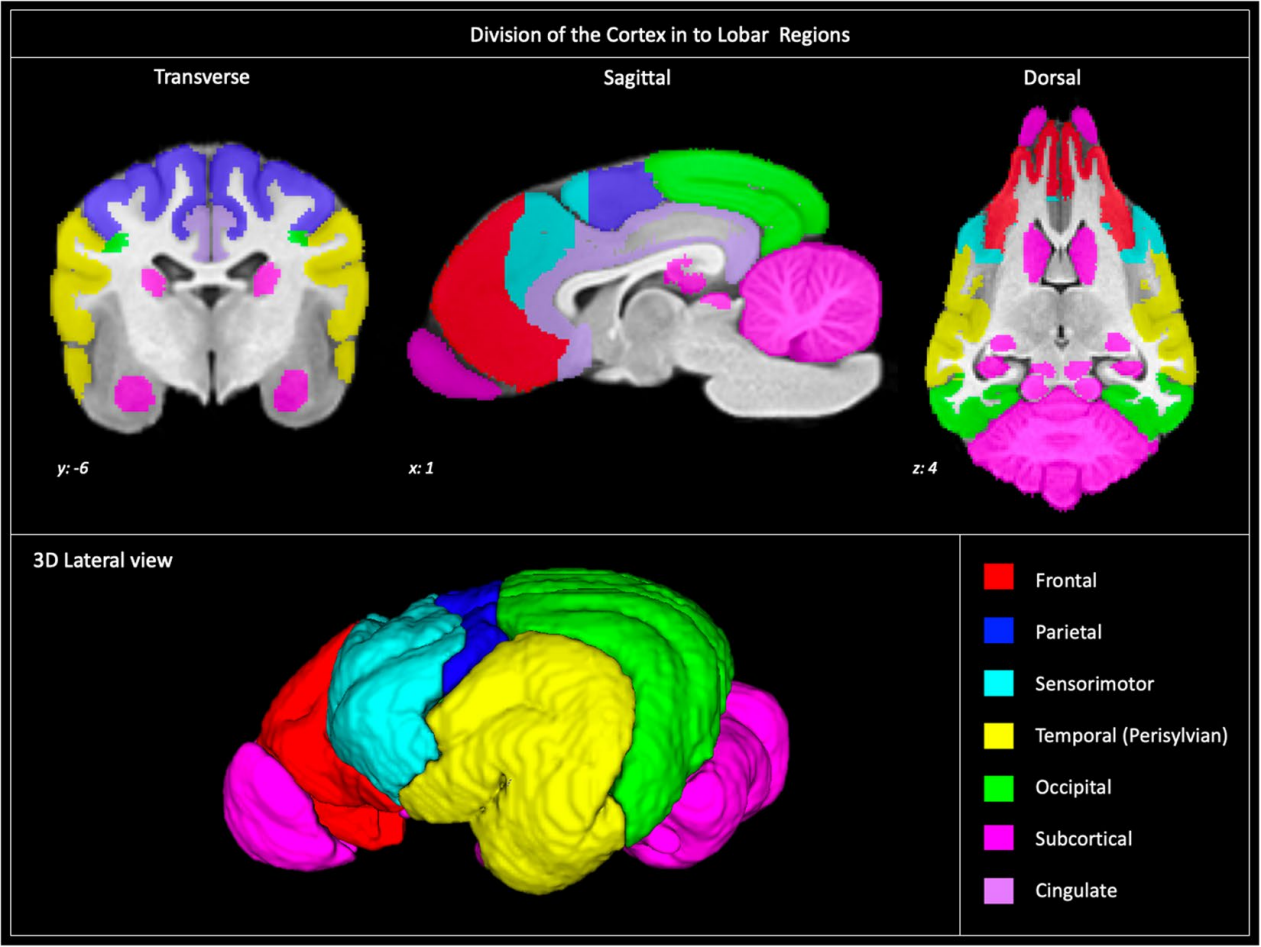
Jaccard similarity after aligned, linear and non-linear registration: Provides a visual demonstration of the overlap of an individual subject’s brain data to the population average template (red outline) after alignment, linear and non-linear registration. A single sample subject from each cranial conformation group is provided. The mean similarity index for each subject was plotted in each cranial conformation group, according to registration technique (aligned = yellow, linear = blue, and non-linear = red). A post hoc Tukey multiple comparisons of means identified statistically significant difference in similarity index between aligned and linear and aligned and non-linear techniques in the brachycephalic group and between aligned and non-linear techniques in the mesaticephalic group. *Jacobian warping after non-linear registration*: Provides a surface heat map (range 0.0–0.8) demonstrating the degree of warping for a single representative subject for each cranial conformation group. The warping metric used is the log demeaned absolute Jacobian determinant for each voxel. The mean Jacobian warping metric for each subject was plotted within each cranial conformation group in the boxplot on the right side. These figures demonstrate that the highest degree of warping was present within the brachycephalic group. This figure was created using FSLeyes (version 2.1 https://fsl.fmrib.ox.ac.uk/fsl/fslwiki/FSLeyes), microGL (version 2.1 www.mricro.com) and Microsoft Powerpoint (version 16.16.19. www.microsoft.com).

## Results

### Template

The final population template exhibited surface detail that corroborated well with an anatomic specimen (Figure 2). Generated tissue segmentation maps exhibited appropriate anatomic structure and correlated well to the grey and white matter definition of the temple.

**Figure 4:**
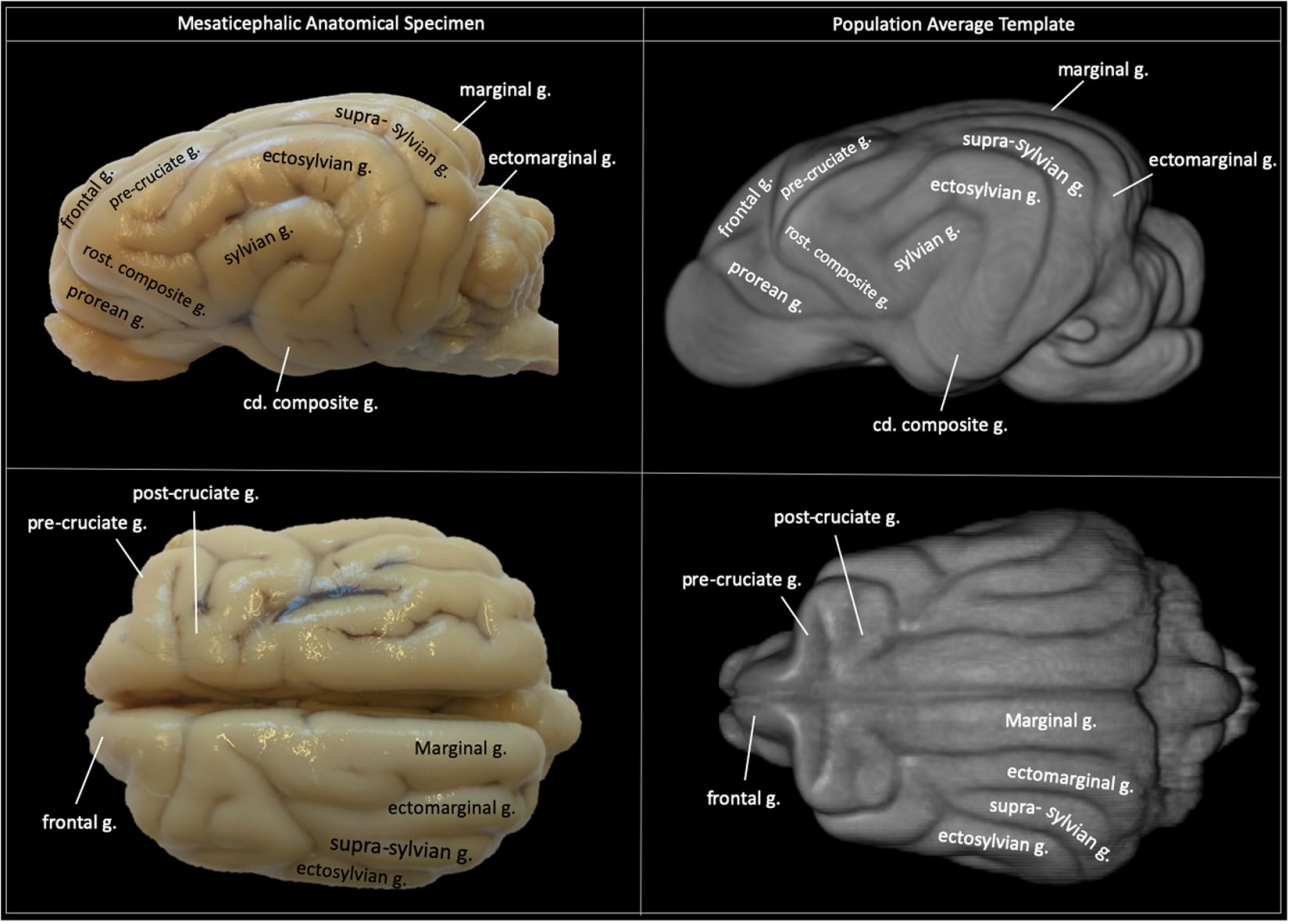
Lobar divisions: Depicts how the brain was divided into lobar regions according to that described by Jerzy Kriener. These regions included frontal (red), parietal (blue), sensorimotor (cian), temporal (yellow), occipital (green), cingulate (mauve), and subcortical (pink). This figure was created using FSLeyes (version 2.1 https://fsl.fmrib.ox.ac.uk/fsl/fslwiki/FSLeyes), ITKsnap (version 3.8.0 www.itksnap.org), Affinity designer (version 1.8 www.affinity.serif.com) and Microsoft Powerpoint (version 16.16.19. www.microsoft.com).

### Skull conformation compatibility

#### Jaccard similarity index

For each skull group, a one-way ANCOVA tested the differences between similarity metrics across registration techniques while controlling for interaction effects of body weight (kg), brain volume (mm^3^) and brain length. Within the brachycephalic group there was significant difference in similarity metrics across registration techniques while controlling for covariates mentioned above (F(2,12) = 144.58, p < 0.001). Post hoc Tukey multiple comparisons of means at 95% family-wise confidence levels showed a significant difference in similarity metrics between alignment and linear registration (p < 0.01) and alignment and nonlinear registration (p < 0.01) but no significant difference in similarity metrics between linear and nonlinear registration. Within the mesaticephalic group, there was a significant difference in similarity metrics across registration techniques while controlling for covariates mentioned above (F(2,8) = 5.29, p = 0.03). Post hoc Tukey multiple comparisons of means at 95% family-wise confidence levels showed a significant difference in similarity metrics between alignment and nonlinear registration (p = 0.04) (Figure 3).

#### Jacobian warping metric

The one samples t-test tested the variation in warping metrics in each skull group. Within the brachycephalic group there appeared to be high levels of warping in the frontal and olfactory cortices, and a large cluster of significant voxels survived multiple comparison correction and 0.95 thresholding. While we observed variations in warping in the mesaticephalic group, there were no significant clusters that survived correction. Variation in localization and magnitude was present across the three representative subjects for brachycephalic, mesaticephalic, and dolichocephalic skull shape (Figure 3).

### Cortical and subcortical parcellation

**Figure 5:**
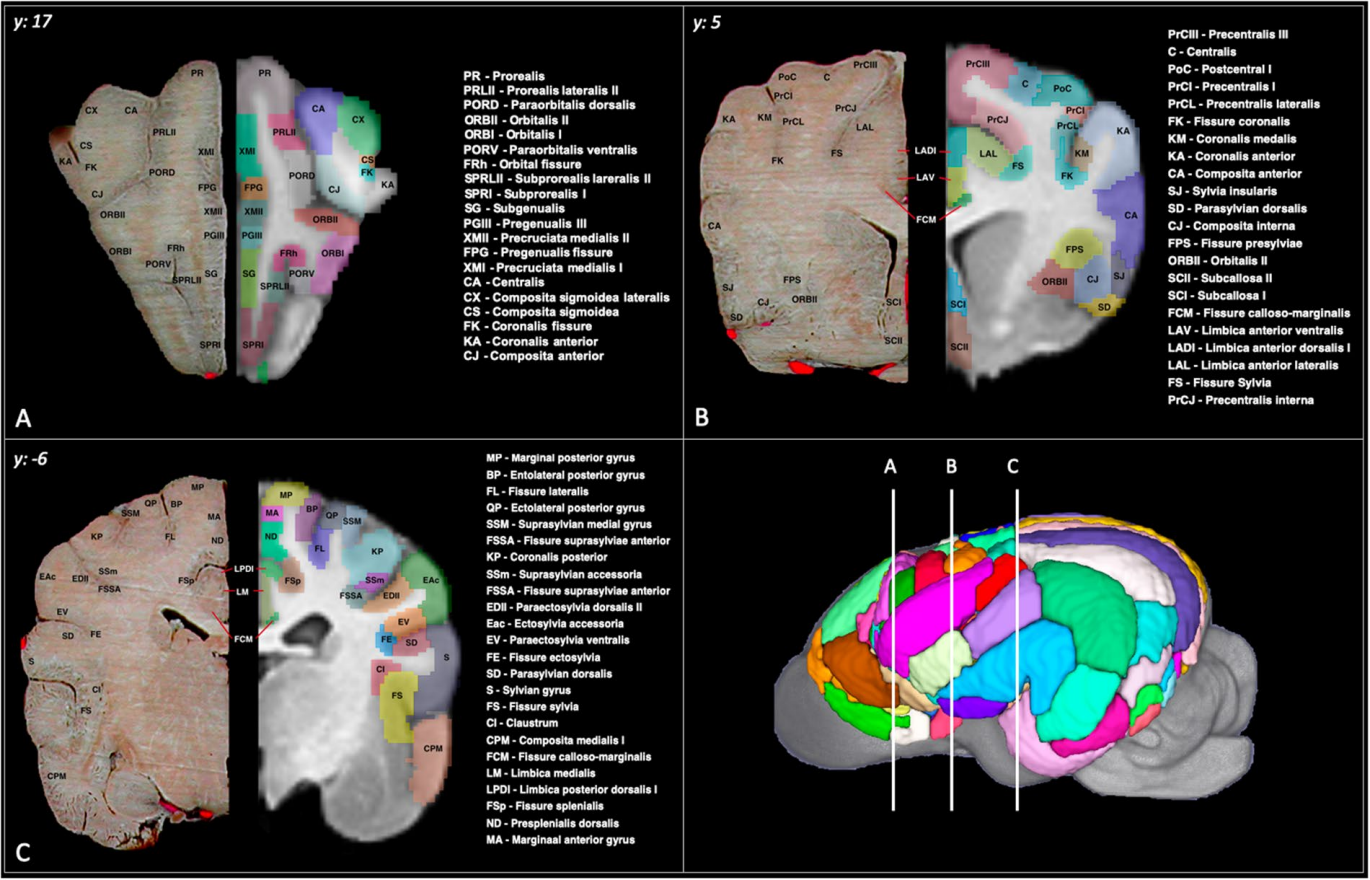
Cortical atlas in transverse sections: Demonstrates the cortical atlas and a corresponding anatomic specimen in transverse section at frontal (**A**), caudate nuclei (**B**) and mid-thalamic (**C**) levels. The anatomic specimen underwent plasticization of the vasculature and fixation. The brain was transected and photographed *in-situ* within the cranium to maintain normal anatomic structure. This figure was created using FSLeyes (version 2.1 https://fsl.fmrib.ox.ac.uk/fsl/fslwiki/FSLeyes), ITKsnap (version 3.8.0 www.itksnap.org), Affinity designer (version 1.8 www.affinity.serif.com) and Microsoft Powerpoint (version 16.16.19. www.microsoft.com).

**Figure 6:**
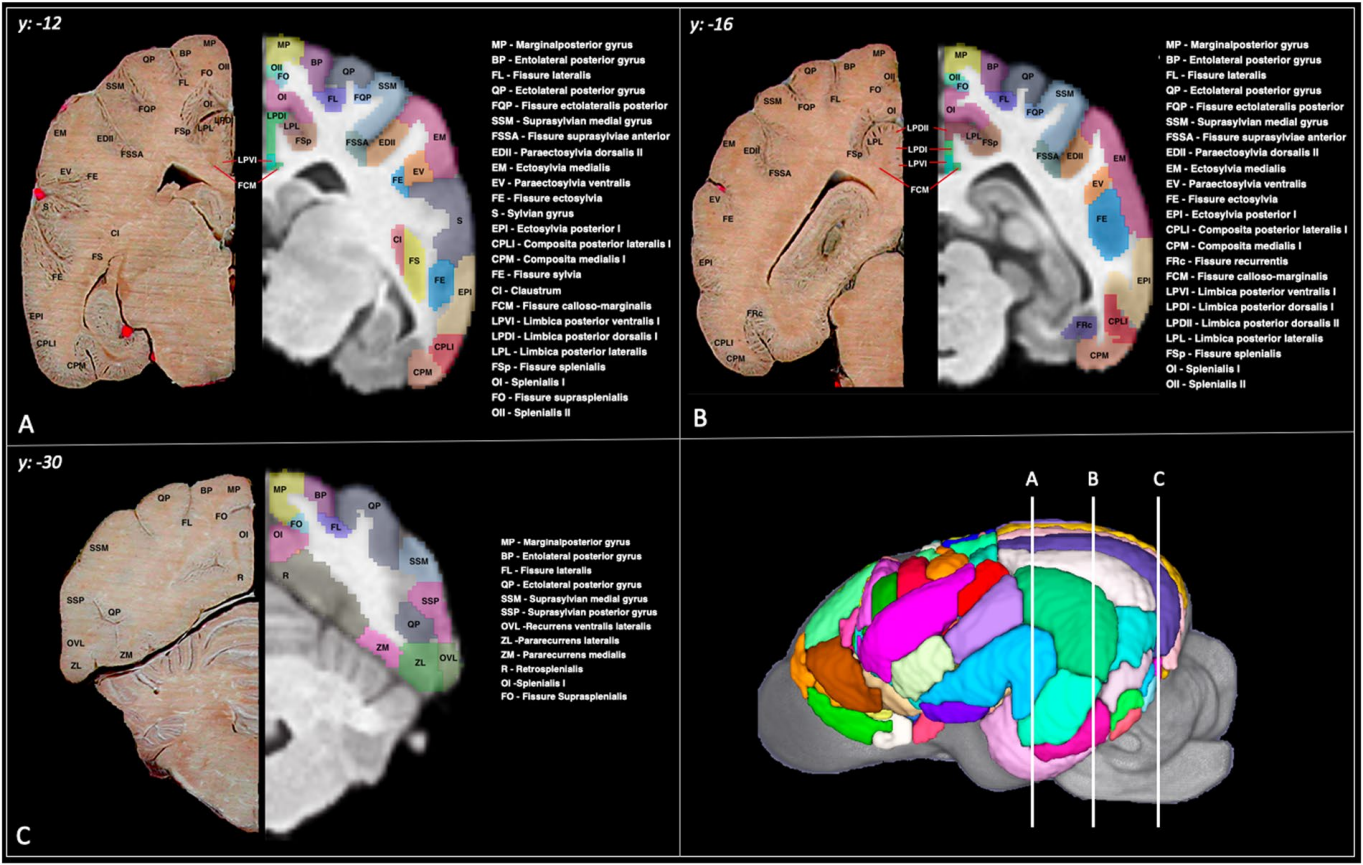
Cortical atlas in transverse sections: Demonstrates the cortical atlas and a corresponding anatomic specimen in transverse section at caudal thalamic (**A**), hippocampal (**B**) and occipital (**C**) levels. The anatomic specimen underwent plasticization of the vasculature and fixation. The brain was transected and photographed *in-situ* within the cranium to maintain normal anatomic structure. This figure was created using FSLeyes (version 2.1 https://fsl.fmrib.ox.ac.uk/fsl/fslwiki/FSLeyes), ITKsnap (version 3.8.0 www.itksnap.org), Affinity designer (version 1.8 www.affinity.serif.com) and Microsoft Powerpoint (version 16.16.19. www.microsoft.com).

**Figure 7:**
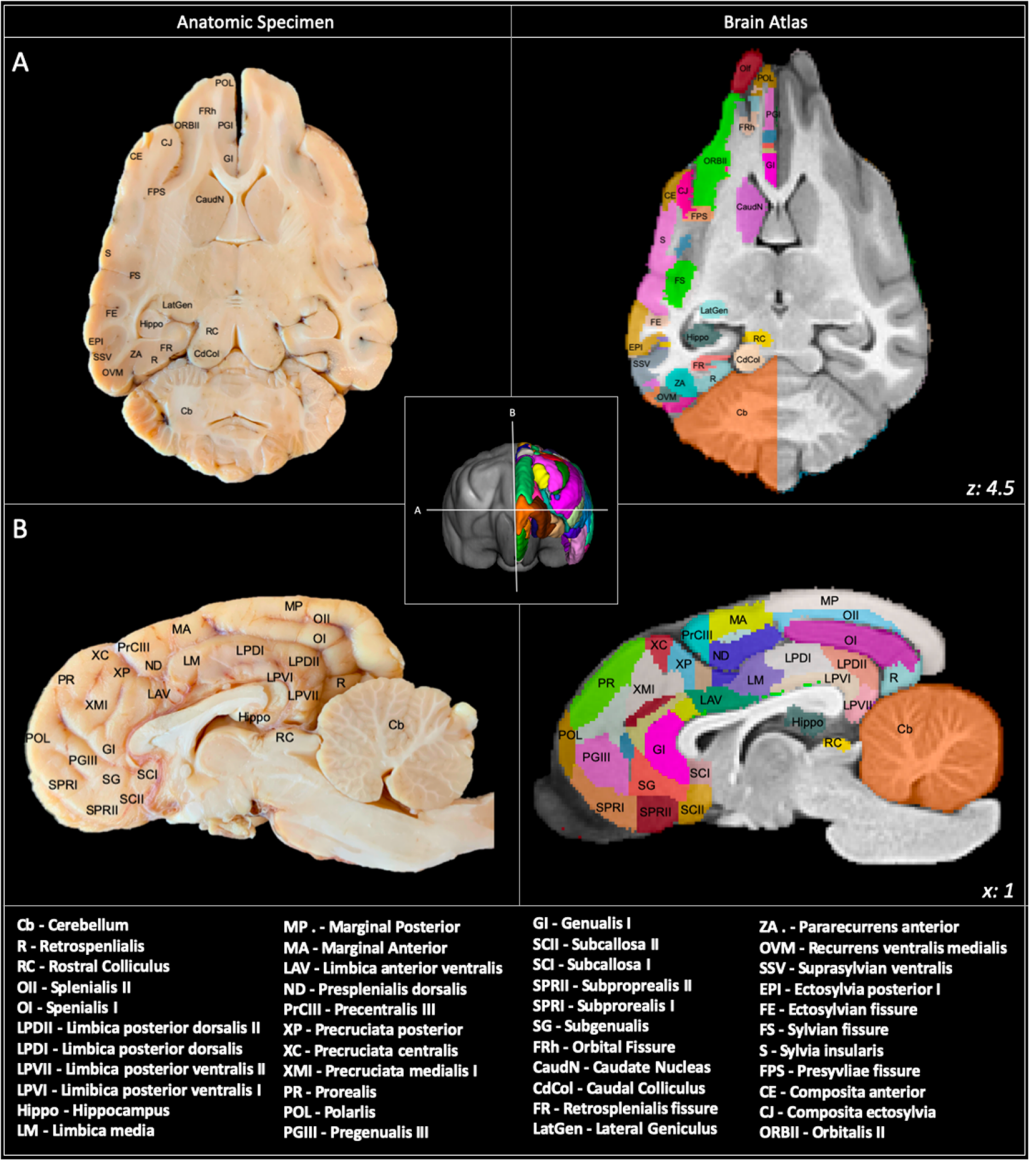
Cortical atlas in sagittal and dorsal sections: Demonstrates the cortical atlas and a corresponding anatomic specimen in dorsal (**A**) and sagittal (**B**) section. The anatomic specimen brain underwent immersion fixation before transection and photography. This figure was created using FSLeyes (version 2.1 https://fsl.fmrib.ox.ac.uk/fsl/fslwiki/FSLeyes), ITKsnap (version 3.8.0 www.itksnap.org), Affinity designer (version 1.8 www.affinity.serif.com) and Microsoft Powerpoint (version 16.16.19. www.microsoft.com).

**Figure 8:**
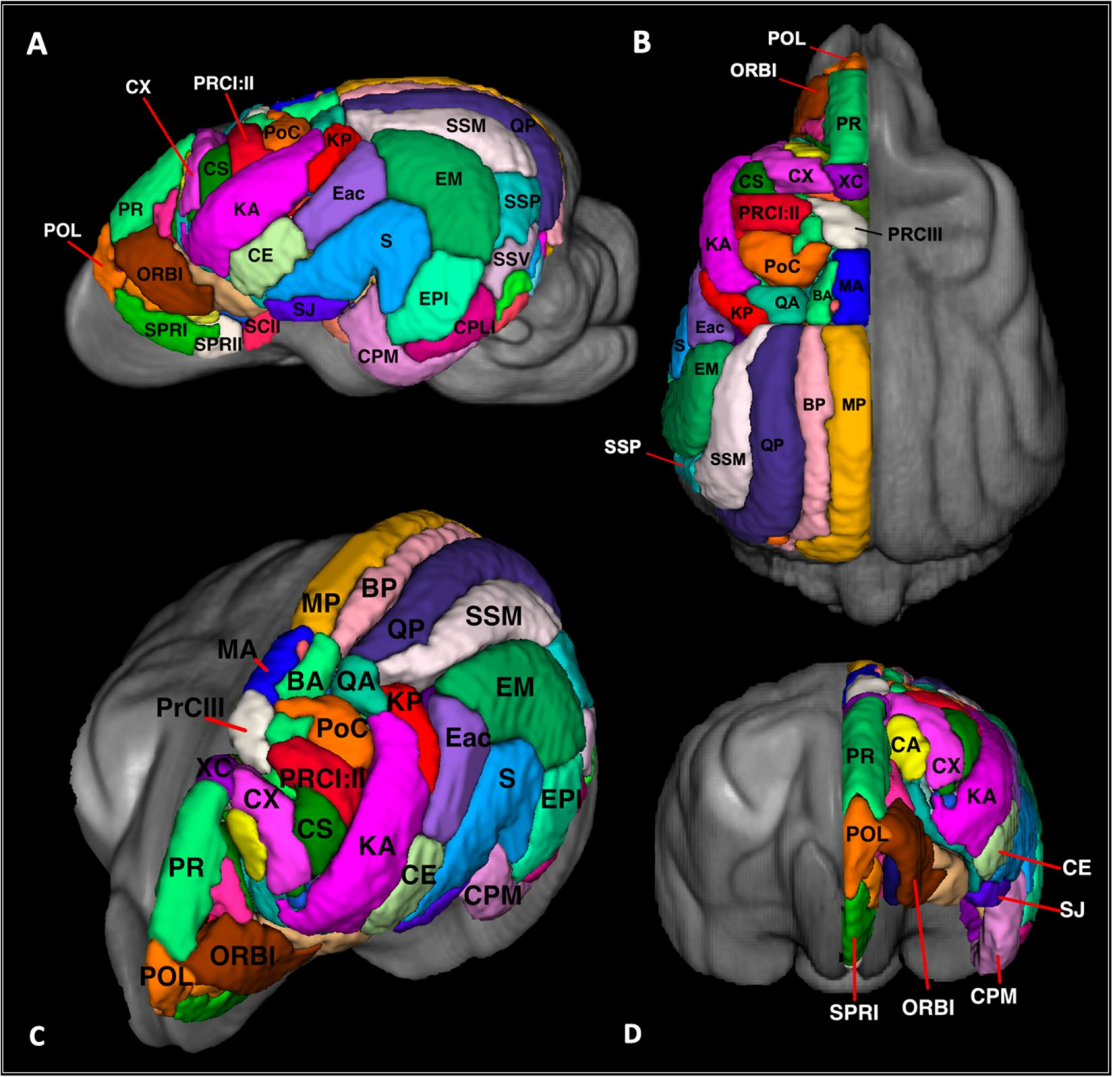
Cortical atlas in 3-dimensions: Demonstrates the 3-dimensional figures of the cortical atlas in lateral (**A**), dorsal (**B**) and oblique (**C**) and frontal (**D**) orientations. This figure was created using FSLeyes (version 2.1 https://fsl.fmrib.ox.ac.uk/fsl/fslwiki/FSLeyes), ITKsnap (version 3.8.0 www.itksnap.org), Affinity designer (version 1.8 www.affinity.serif.com) and Microsoft Powerpoint (version 16.16.19. www.microsoft.com).

The brain was parcellated into seven lobar regions (Figure 4) and a total of 234 cortical and subcortical regions. The abbreviation, full name, gyrus, lobe and volume of each region is documented in Table 3. Transverse, sagittal and dorsal sliced images or the cortical parcellation with anatomic referencing is provided in Figures 5–7 and three -dimensional depictions provided in Figure 8.

**Table 3:** Documents the name, abreviation, gyral and lobar location and volume of each cortical and subcortical prior.

Abbrev.	Full Name	Gyri	Lobe	Left Volume (mm^3^)	Right Volume (mm^3^)
FCM	Area fissurae calloso-marginalis		Cingulate	274	280
GI	Area genualis I	Genualis Gyrus	Cingulate	175	179
GII	Area genualis II	Genualis Gyrus	Cingulate	934	972
LADI	Area limbica anterior dorsalis I	Anterior Cingulate Gyrus	Cingulate	310	310
LADII	Area limbica anterior dorsalis II	Anterior Cingulate Gyrus	Cingulate	195	189
LAL	Area limbica anterior lateralis	Cingulate Gyrus	Cingulate	474	473
LAV	Area limbica anterior ventralis	Anterior Cingulate Gyrus	Cingulate	527	504
LM	Area limbica media	Cingulate Gyrus	Cingulate	819	651
LPDI	Area limbica posterior dorsalis I	Posterior Cingulate Gyrus	Cingulate	1023	968
LPDII	Area limbica posterior dorsalis II	Posterior Cingulate Gyrus	Cingulate	905	828
LPL	Area limbica posterior lateralis	Posterior Cingulate Gyrus	Cingulate	511	496
LPVI	Area limbica posterior ventralis I	Posterior Cingulate Gyrus	Cingulate	599	621
LPVII	Area limbica posterior ventralis II	Posterior Cingulate Gyrus	Cingulate	539	551
SCI	Area subcallosa I	Subcallosus Gyrus	Cingulate	553	569
SCII	Area subcallosa II	Subcallosus Gyrus	Cingulate	216	205
FRh	Area fissurae orbitalis	Orbital Gyrus	Frontal	555	651
ORBI	Area orbitalis I	Orbital Gyrus	Frontal	3353	3170
ORBII	Area orbitalis II	Orbital Gyrus	Frontal	2561	2599
PGI	Area pregenualis I	Pregenual Gyrus	Frontal	150	162
PGII	Area pregenualis II	Pregenual Gyrus	Frontal	983	834
PGIII	Area pregenualis III	Pregenual Gyrus	Frontal	826	807
POL	Area Polaris	Gyrus Proreus	Frontal	784	832
PORD	Area paraorbitalis dorsalis	Orbital Gyrus	Frontal	713	743
PORV	Area paraorbitalis ventralis	Orbital Gyrus	Frontal	563	555
PR	area prorealis	Gyrus Proreus	Frontal	457	464
PRLI	Area Prorealis lateralis I	Gyrus Proreus	Frontal	459	508
PRLII	Area prorealis lateralis II	Gyrus Proreus	Frontal	2838	3042
SG	Area subgenualis	Pregenual Gyrus	Frontal	737	617
SPRI	Area Subprorealis I	Gyrus Subproreus	Frontal	664	617
SPRII	Area Subprorealis II	Gyrus Subproreus	Frontal	1343	1376
SPRLI	Area Subprorealis lateralis I	Gyrus Subproreus	Frontal	272	274
SPRLII	Area Subprorealis Lateralis II	Gyrus Subproreus	Frontal	436	495
BP	Area entolateralis posterior	Entolateral Gyrus	Occiptal	4891	4818
FL	Area fissurae lateralis		Occiptal	2173	1993
FO	Area fissurae suprasplenialis	Marginal Gyrus	Occiptal	732	577
FQ	Area fissurea ectolateralis	Ectolateral Gyrus	Occiptal	1004	830
FQP	Area fissurea ectolateralis posterior	Ectolateral Gyrus	Occiptal	608	659
FR	Area fissurea retrosplenialis	Medial Occipital Gyrus	Occiptal	3164	3199
FRc	Area fissurea recurrentis	Medial Occipital Gyrus	Occiptal	1591	1346
FSp	Area fissurea splenialis	Medial Occipital Gyrus	Occiptal	1762	1588
FSSA	Area fissurea suprasylviae anterior	Suprasylvian Gyrus	Occiptal	2123	2075
MP	Area marginalis posterior	Marginal Gyrus	Occiptal	7131	7710
OI	Area splenialis I	Marginal Gyrus	Occiptal	926	859
OII	Area splenialis II	Marginal Gyrus	Occiptal	3669	3769
ORL	Area recurrens lateralis	Recurrens	Occiptal	464	548
ORM	Area recurrens medialis	Recurrens	Occiptal	735	756
OVL	*Area recurrens ventralis lateralis*	Recurrens	Occiptal	324	294
OVM	*Area recurrens ventralis medialis*	Recurrens	Occiptal	419	382
QP	Area ectolateralis posterior	Ectolateral Gyrus	Occiptal	7130	7541
R	Area retrospenialis	Medial Occipital Gyrus	Occiptal	4523	4308
SSM	Area suprasylvian medialis	Suprasylvian Gyrus	Occiptal	7012	7392
SSP	Area suprasylvian posterior	Suprasylvian Gyrus	Occiptal	3871	3483
SSV	Area suprasylvian ventralis	Suprasylvian Gyrus	Occiptal	1246	1356
ZA	Area pararecurrens anterior	Pararecurrens Gyrus	Occiptal	1423	1549
ZL	Area pararecurrens lateralis	Pararecurrens Gyrus	Occiptal	430	484
ZM	Area pararecurrens medialis	Pararecurrens Gyrus	Occiptal	320	266
BA	Area entolateralis anterior	Entolateral Gyrus	Parietal	236	223
BAL	Area entolateralis anterior lateralis	Entolateral Gyrus	Parietal	717	684
FA	Area fissurae ansata	Marginal Gyrus	Parietal	284	313
FBA	Area fissurae entolateralis pars anterior	Entolateral Gyrus	Parietal	52	42
FL	Area fissurae lateralis	Entolateral Gyrus	Parietal	304	244
FN	Area fissurae suprasplenialis	Marginal Gyrus	Parietal	154	153
FSPL	Area fissurae presylviae lateralis	Marginal Gyrus	Parietal	471	463
KP	Area coronalis posterior	Coronal Gyrus	Parietal	351	359
KPL	Area coronalis posterior lateralis	Coronal Gyrus	Parietal	622	690
KPM	Area coronalis posterior medialis	Coronal Gyrus	Parietal	1515	1503
MA	Area marginalis anterior	Marginal Gyrus	Parietal	1283	1339
ML	Area marginalis lateralis	Marginal Gyrus	Parietal	198	161
ND	Area presplenialis dorsalis	Presplenial Gyrus	Parietal	1226	1252
NV	Area presplenialis ventralis	Presplenial Gyrus	Parietal	431	405
QA	Area ectolateralis anterior	Ectolateral Gyrus	Parietal	813	718
SSm	Area suprasylvian accessoria	Suprasylvian Gyrus	Parietal	106	123
CI	Centralis I	Pre/postcentral Gyrus	Temporal	2177	1902
CPLI	Area composita posterior lateralis I	Posterior Compositus Gyrus	Temporal	4397	3986
CPM	Area composita medialis I	Posterior Compositus Gyrus	Temporal	727	844
Eac	Area ectosylvia accessoria	Ectosylvian Gyrus	Temporal	2301	2466
EDII	Area paraectosylvia dorsalis II	Ectosylvian Gyrus	Temporal	3973	3565
EM	Area ectosylvia medialis	Ectosylvian Gyrus	Temporal	5434	5741
EPI	Area ectosylvia posterior I	Ectosylvian Gyrus	Temporal	2990	3148
EV	Area paraectosylvia ventralis	Ectosylvian Gyrus	Temporal	1879	1892
FE	Area fissurae ectosylvia	Ectosylvian Gyrus	Temporal	2220	2366
FS	Area fissurae sylvia	Sylvian Gyrus	Temporal	2760	2604
S	Area sylvia	Sylvian Gyrus	Temporal	1100	912
SD	Area parasylvian dorsalis	Sylvian Gyrus	Temporal	1624	1452
SJ	Area sylvia insularis	Sylvian Gyrus	Temporal	6092	6572
C	Area centralis	Central Gyrus	Sensory-motor	569	588
CA	Area composita anterior	Anterior Compositus Gyrus	Sensory-motor	2251	2663
CE	Area composita ectosylvia	Anterior Compositus Gyrus	Sensory-motor	2783	2746
CJ	Area composita interna	Anterior Compositus Gyrus	Sensory-motor	364	375
CS	Area composita sigmoid	Anterior Compositus Gyrus	Sensory-motor	990	1117
CSL	Area composita sigmoidea lateralis	Anterior Compositus Gyrus	Sensory-motor	1724	1660
CX	Area composita precruciata	Precruciate Gyrus	Sensory-motor	365	356
FK	Area fissurae coronalis	Coronal Gyrus	Sensory-motor	1419	1469
FPG	Area fissurae pregenualis	Coronal Gyrus	Sensory-motor	372	372
FPS	Area fissurae presylviae	Anterior Compositus Gyrus	Sensory-motor	1345	1375
FS	Area fissurae splenialis	Precruciate Gyrus	Sensory-motor	1038	929
KA	Area coronalis anterior	Coronal Gyrus	Sensory-motor	5189	4987
KM	Area coronalis medialis	Coronal Gyrus	Sensory-motor	717	600
PoC	Area postcentralis I	Postcentral Gyrus	Sensory-motor	1803	1976
PrCI/II	Area precentralis I/II	Precentral Gyrus	Sensory-motor	1532	1462
PrCIII	Area precentralis III	Precentral Gyrus	Sensory-motor	1124	1230
PrCJ	Area precentralis interna	Precentral Gyrus	Sensory-motor	1244	1246
PrCL	Area precentral lateralis	Precentral Gyrus	Sensory-motor	306	268
XC	Area precruciata centralis	Precruciate Gyrus	Sensory-motor	730	799
XL	Area precruciata lateralis	Precruciate Gyrus	Sensory-motor	766	732
XMI	Area precruciata medialis I	Precruciate Gyrus	Sensory-motor	642	611
XMII	Area precruciata medialis II	Precruciate Gyrus	Sensory-motor	1111	1046
XP	Area precruciata posterior	Precruciate Gyrus	Sensory-motor	649	620
Amyg	Amygdala	Subcortical Regions	Subcortical	1228	1148
CaudN	Caudate Nucleas	Subcortical Regions	Subcortical	5195	5100
CdColl	Caudal Colliculus	Subcortical Regions	Subcortical	929	964
Cere	Cerebellum	Subcortical Regions	Subcortical	39456	39058
Hippo	Hippocampus	Subcortical Regions	Subcortical	5625	5937
LatGen	Lateral Geniculate	Subcortical Regions	Subcortical	483	554
MedGen	Medial Geniculate	Subcortical Regions	Subcortical	333	330
Olf	Olfactory Bulb	Olfactory Bulbs	Subcortical	8810	8337
RostColl	Rostral Colliculus	Subcortical Regions	Subcortical	411	426

### Frontal parcellation

The frontal region was delineated by adapting from what Brodmann termed the “regio frontalis” in man^14^ and was bordered ventrally by the anterior rhinal sulcus and caudally by the sylvian and genual sulci^25^. This region involved the orbital, pregenual, proreus and subproreus gyri and was segmented into 17 different regions per hemisphere. These regions had a mean volume of 1042.4 mm^3^ (+/−918.1) (Table 3).

### Sensori-motor parcellation

The sensori-motor region was delineated according to that described by Woosley and his associates^45^ and includes the pre-cruciate, anterior composite, precentral, postcentral and coronal gyri^26^. This region was segmented into 23 different regions per hemisphere. These regions had a mean volume of 1266.5 mm^3^ (+/−1037.0) (Table 3).

### Cingular parcellation

The cingular cortex represents the limbic region in the dog and comprises subcallosal, genual, anterior cingulate and posterior cingulate gyri. It lies adjacent to the callosal commissure and is borders the deep fissure splenialis dorsolaterally, genual fissure rostrally and caloso-marginalis fissure ventrally^24^. This region was segmented into 15 different regions per hemisphere. These regions had a mean volume of 528.3 mm^3^ (+/−261.5) (Table 3).

### Parietal parcellation

The parietal region lies caudal to the sensori-motor cortex and is bordered by the splenial fissure medially and suprasylvian fissure laterally. This area includes regions of the entolateral, marginal, coronal, presplenial, ectolateral and suprasylvian gyri and is divided into 16 regions per hemisphere^23^. These regions had a mean volume of 544.8 mm^3^ (+/−440.3) (Table 3).

### Temporal (peri-sylvian) parcellation

This region lies laterally and includes the sylvian, ectosylvian and posterior composite gyri and functionally represents the auditory cortex^28^. This area is divided into 13 different hemispheric regions. These regions had a mean volume of 2889.4 mm^3^ (+/−1618.3) (Table 3).

### Occipital parcellation

This region lies caudally within the brain and its margin borders the posterior rhinal, retrosplenial and posterior suprasylvian fissures. It includes regions within the entolateral, marginal, ectolateral, medial occipital, suprasylvian, recurrens and pararecurrents gyri^27^. This area was segmented into 24 different regions according to the myeloarchitectonic structure. These regions had a mean volume of 2405.2 mm^3^ (+/−2274.3) (Table 3).

### Subcortical parcellation

These regions were delineated according to anatomic descriptions^43^ and included the amygdala, caudate nuclei, rostral and caudal colliculus, cerebellum, hippocampi, lateral and medial geniculate nuclei and olfactory bulbs. We included only regions whose boundaries were readily visible on the T1-weighted atlas were included in these segmentations. These regions had a mean volume of 6906.9 mm^3^ (+/−11776.5) (Table 3).

### Using this brain atlas

This atlas can be used with common MRI toolboxes such as FSL (https://fsl.fmrib.ox.ac.uk/fsl/fslwiki) and ANTs (http://stnava.github.io/ANTs/) to perform linear or nonlinear registration from subject’s T1 native space to the atlas T1 population space or, inversely, to register T1 population template to a subject’s T1 native space. The authors would suggest using either FSL’s FLIRT (https://fsl.fmrib.ox.ac.uk/fsl/fslwiki/FLIRT) for linear registration or ANTs SyN^35^ for nonlinear registration, saving the transformation matrices of these registrations and applying them to the brain atlas or other masks. Visual or manual registration can be conducted with itk-SNAP^46^ if necessary or desired. To view the atlas with labels users can use FSLeyes (https://zenodo.org/record/3530921#.Xkbq1hdKhUM). Once the atlas is loaded the atlas search tab can be used to identify and isolate specific regions by label name.

### Ethics statement

All animal use associated with this study was approved by institutional ethics or animal care and use committees.

## Discussion

We present a comprehensive cortical atlas for the canine brain based on cortical myeloarchitecture. This atlas includes a population average template generated from 30 neurologically normal non-brachycephalic canines and TSMs for GM, WM and CSF. Cortical parcellation resulted in the generation of 234 cortical and subcortical priors from frontal, sensorimotor, parietal, temporal (perisylvian), occipital, cingular and subcortical regions. Non-linear registration of canine brains from mesaticephalic, dolichocephalic and brachycephalic cranial conformation resulted in high levels of similarity but significant warping within the brachycephalic group. The atlas is made available through an online repository https://ecommons.cornell.edu/handle/1813/67018.

### Importance of this brain atlas

This is the most comprehensive architectonically parcellated cortical atlas created for the dog, an essential neuroscientific animal model. Modern stereotaxic brain atlases are a vital tool for neuroimaging research with far-reaching applications in data normalization, registration, segmentation and parcellation^47^. The lack of a detailed cortical atlas has, so far, limited researchers working with the dog model^3^. Although an increasing number of studies perform fMRI on the awake and anesthetized canines, the lack of an accepted high-quality canine atlas has limited group-level and cortical region of interest analyses^2,48–52^. Our atlas is a vital tool that will help standardize cortical localization of regions of functional activation improving our understanding of the functional-structural correlation of the canine brain.

Analyzing the resting-state default mode network is a promising area of research in the canine^1^. However, as yet, only independent component analysis (ICA) and manually placed seed-based analysis have been performed^52^. Our atlas provides whole-brain architectonic based cortical priors that could standardize seed-based functional connectivity analysis and assist in interpreting ICA. Vogt and Vogt suggested that the unique nature of each cortical region’s myeloarchitectonic structure indicated that every region has a separate and specific function^53^. fMRI has helped to identify specific regions of the brain that respond to different stimuli, including audition^54^, olfaction^50,55^ and visual facial processing^48^. Correlating these findings to our cortical brain atlas could help define the functional relevance of these architectonically distinct regions, taking us a step further in understanding the structure-function relationship of the canine brain and how this correlates to what is already well established in humans.

Cortical parcellation can be performed using multiple methods, including architectonics, surface structure, connectivity, electrophysiology and function. The paucity of functional, electrophysiological and connection data for the dog precluded the use of these techniques to create a comprehensive cortical atlas. Architectonic based cortical parcellation has historically created the most important and readily used atlases in the human^14^ and multiple animal models^56,57^. Architectonics uses cellular structure and organization to delineate boundaries within the cortex and includes both cytoarchitectonic and myeloarchitectonic methods. In the dog comprehensive histology-based atlases have been created using both cytoarchitectonic and^58–60^ and myeloarchitectonic^23–28^ techniques. The cytoarchitectonic based atlases are relatively simple, exhibit considerable variation in cortex partitioning, and lack cross-sectional illustrations. Thus, making accurate delineation of cortical regions throughout the complex canine brain extremely challenging^58–60^. Also, fMRI research raises the concern that cytoarchitectonic based atlases underestimate the degree of cortical partitioning at a functional level^16,61,62^. For these reasons, we created our cortical atlas with guidance from the comprehensive series of papers documenting cortical parcellation according to myeloarchitectonic structure by Jerzy Kreiner^23–28^.

Kreiner divided the cortex by assessing the size, staining, appearance, and arrangement of radial and tangential fibers and the appearance of fibers in the superficial plexus^24^. Myeloarchitectonic based cortical parcellation was the initial technique used to divide the human cortex by the anatomists Cecil and Oskar Vogt^20^. This technique is thought to corroborate with cytoarchitectonic based cortical divisions and has been used to create a cortical “supermap” in man^16,20^. When Kreiner compared his myeloarchitectonic cortical division of the canine brain to atlases using cytoarchitectonic based parcellation, there were both similarities and apparent differences in parcellation of the cortex between techniques^23–28,58–60^. In the human brain, parcellation similarly identified disparities between the Vogt-Vogt myeloarchitectonic atlas and the cytoarchitectonic-based Brodmann atlas. However, when Vogt and Vogt, and multiple other researchers combined these techniques, they described complete concordance between cytoarchitectonic and myeloarchitectonic based regions^20,63–65^.

Myeloarchitectonic cortical parcellation identifies boundaries within the cortex according to the organization and structure of myelinated fiber layers and radial bundles^19^. Myelin has a specific signal intensity on MRI and recently non-invasive imaging techniques have been used to create cortical myelin maps *in vivo*. These techniques take advantage of the intensity differences between degrees of myelination within grey matter observed on T1 and T2 weighted sequences and create cortical myelin maps with distinctive patterns of light, moderate and heavy myelination^66^. These *in vivo* maps have been found to correlate well with both cytoarchitectonic and myeloarchitectonically defined cortical boundaries^16,66–68^. *In vivo* cortical myelin maps have not, as yet, been generated for the canine and our atlas serves as a useful tool for validation and interpretation of future study in this area.

It is optimal to utilize an atlas that most closely resembles the brain structure of the study population^47^. Dogs have highly variable brain structure depending on their cranial conformation and breed^69,70^. Most importantly, brachycephalic dogs exhibit shortening of the cranium that causes ventral pitching of the brain’s long-axis and a ventral shift of the olfactory lobe^69^. The degree of brain deformity associated with brachycephaly warrants a specific brachycephalic population template, as is provided by Milne *et al*.^11^. With this in mind, we limited differences in brain structure within our template cohort by including only dogs with mesaticephalic or dolichocephalic cranial conformation and excluding brachycephalics. As a result, our atlas is most suitable for non-brachycephalic canine cohorts, which includes the most common pet dog breeds, the golden retriever, Labrador retriever, German shepherd dog, and the most commonly used research dog breed, the beagle. When we tested the effect of registration of subjects with brachycephalic cranial conformation to the final template, we found that although non-linear registration resulted in a high degree of similarity between the template and the subject, there was an associated high level of data warping. Excessive degrees of warping can create artifact and misclassification of tissues and structures^47,71^. Considering this limitation is essential when using this atlas in populations of dogs with brachycephalic cranial conformation. The development of parcellated cortical atlases specific to dogs with brachycephaly cranial conformation could be a focus of further study.

The dog is becoming an increasingly important animal model for neurocognitive, translational and comparative neuroscience research; however, tools such as a cortical brain atlas, are required to support research in this species^3^. We generated this cortical brain atlas from high-quality isovolumetric T1-weighted data obtained from 30 neurologically and clinically healthy dogs. It includes a population average template, tissue probability maps and 234 cortical and subcortical priors from frontal, sensorimotor, parietal, temporal (perisylvian), occipital, cingular and subcortical regions. The resulting population template has been validated using additional populations of mesaticephalic, brachycephalic and dolichocephalic skull conformations. This atlas will improve tissue segmentation and cortical region delineation and represents a unique and vital tool to facilitate neuroimaging research in this useful animal model.

## Data Availability

The presented data set are stored in NIFTI-1 format and can be viewed on readily available imaging software including SPM and FSL (Analysis Group, FMRIB, Oxford, UK). All data including the T1-weighted population average canine brain template, cortical and subcortical priors, tissue segmentation maps are available at the following online resource center https://ecommons.cornell.edu/handle/1813/67018.
