# Publisher Correction: Stereotactic Cortical Atlas of the Domestic Canine Brain

**DOI:** 10.1038/s41598-021-84559-1

**Published:** 2021-02-23

**Authors:** Philippa J. Johnson, Wen-Ming Luh, Benjamin C. Rivard, Kathleen L. Graham, Andrew White, Marnie FitzMaurice, John P. Loftus, Erica F. Barry

**Affiliations:** 1 Cornell College of Veterinary Medicine, Department of Clinical Sciences, Cornell University, Ithaca, NY USA; 2 National Institute of Aging, National Institutes of Health, Baltimore, MD USA; 3 Clinical Ophthalmology and Eye Health, Sydney Medical School, University of Sydney, Sydney, NSW Australia; 4 Cornell College of Veterinary Medicine, Department of Biomedical Sciences, Cornell University, Ithaca, NY USA

Correction to: *Scientific Reports* 10.1038/s41598-020-61665-0, published online 16 March 2020

The original version of this Article contained a typographical error in the spelling of the author Marnie FitzMaurice, which was incorrectly given as Marnie Fitz-Maurice.

Additionally, in the original version of this Article, the Author Contribution section was omitted.

These errors has now been corrected in the HTML and PDF versions of the Article.

Lastly, the original HTML version of this Article contained an error in the order of the Figures. Figures 2, 3 and 4 were published as Figures 4, 2, and 3 respectively. As a result, the Figure legends were incorrect. These errors have now been corrected in the HTML version of the Article; the PDF version was correct from the time of publication.

